# Heart Protective Effects of Electroacupuncture in an Animal Experimental Study with Delayed Fluid Resuscitation after Hemorrhagic Shock

**DOI:** 10.1155/2018/2513791

**Published:** 2018-04-02

**Authors:** Huan Wang, Zhen Liu, Yuanshi Liu, Zhanqi Tong, Yan Qian, Liping Chen, Bin Jiang, Mingxiong Lin, Tao Yang, Lu Gao, Mingjun Chen, Daniela Litscher, Lu Wang, Gerhard Litscher

**Affiliations:** ^1^Department of TCM and Acupuncture, People's Liberation Army General Hospital, Beijing 100853, China; ^2^TCM Research Center Graz, Research Unit of Biomedical Engineering in Anesthesia and Intensive Care Medicine and Research Unit for Complementary and Integrative Laser Medicine, Medical University of Graz, 8036 Graz, Austria; ^3^Department of Gastroenterology, Guang'anmen Hospital, China Academy of Chinese Medical Sciences, Beijing 100053, China; ^4^Department of Acupuncture, Guang'anmen Hospital, China Academy of Chinese Medical Sciences, Beijing 100053, China

## Abstract

Fluid resuscitation could hardly be performed immediately after fatal hemorrhagic shock in outpatients. We investigated whether electroacupuncture (EA) at Zusanli (ST36) could prevent fatal hemorrhagic shock induced heart failure with delayed fluid resuscitation and whether the protective role of EA is related to the autonomic nervous system. Sixty Sprague Dawley rats were randomly divided into five groups (*n* = 12 each): group of sham hemorrhagic shock (SHAM), group of EA, group of sham EA (SEA), group of delayed fluid resuscitation with EA (EA + DR), and group of delayed fluid resuscitation with SEA (SEA + DR). After blood loss for 6 hours, caspase-3 activity and positive rate of TUNEL in EA + DR group were significantly lower than in other hemorrhagic shock groups (e.g., versus SEA + DR: 0.156 ± 0.039 versus 0.301 ± 0.042; *P* < 0.05). Immediately EA treatment after the blood loss enhanced the protective effect of delayed resuscitation on the cardiac tissue of hemorrhagic shock rats. Considering the significant changes of epinephrine (137.8 ± 6.9 ng/L versus 98.6 ± 7.4 ng/L; *P* < 0.05) and acetylcholine (405 ± 8.6 pmol/L versus 341 ± 10.1 pmol/L; *P* < 0.05) after EA treatment (SEA + DR versus EA + DR), this cardiac protective effect may be related to regulation of the autonomic nervous system.

## 1. Introduction

Hemorrhagic shock (HS) is one of the most important causes of early mortality in trauma patients [1]. Current guidelines for the treatment of HS recommend that fluid replacement using warmed isotonic electrolyte solutions should be started immediately after the event [2]. However, blood products may not always be immediately available; therefore, additional methods and administrations may be required.

Sympathetic overactivity in the early stage of shock leads to tissue ischemia and oxygen deficit, which produce massive CO_2_ and lactic acid and backflow into blood, causing acidosis. In the meantime, metabolic disorders can create various pathological products, such as tumor necrosis factor (TNF) and interleukin-1 (IL-1). The above-mentioned pathological products can directly influence the coupling between adenylate cyclase and adrenergic receptor, reducing the adrenergic recipient affinity, and even directly influence the amount of recipients [3, 4]. The responsiveness of the recipient on smooth muscle to catecholamine can cause angiectasis and sustained hypotension. The progressive decrease in arterial blood pressure and the decrease in effective blood perfusion in the body of patients with hemorrhagic shock will accelerate the progress of the disease and worsen the functional metabolic disorder, which leads to the nonfunction of significant organs such as the heart, causing irreversible damage. Research [5, 6] indicates that EA at ST36 can promote periphery nerve endings producing acetylcholine, enhancing the activity of acetylcholine, and activating the cholinergic anti-inflammatory pathway. It has positive therapeutic implications on a series of inflammatory reactions that occur during shock, protecting the functions of organs of the body after shock [7]. One of our previous studies [8] showed that the ST36 acupuncture point improved rehydration and rats' blood pressure levels and decreased the survival rate of rats in hemorrhagic shock. Function and structure of cardiac tissue are one of the key factors affecting prognosis of hemorrhagic shock. EA treatment could improve the blood pressure of hemorrhagic rats and decrease the risk of rats' hemorrhagic shock mortality. Therefore we assume that EA treatment could have a heart protective effect when rats are suffering a hemorrhagic shock.

There are many researches about the EA anti-inflammatory effect but few on the EA heart protective effects [9–11]. So, in this study, we observed the cardiac apoptotic nuclei of each group under fluorescence microscope and calculated the number of them by TUNEL assay. For more accurate expression, caspase-3 protein activity assay was also be used to test the cardiac function and structure. Acupuncture as one of the therapeutic maneuvers in Traditional Chinese Medicine has been applied for thousands years and some of its basic mechanism remains still unknown. In one of our previous studies [12] we have proven that EA treatment could activate the cholinergic anti-inflammatory-dependent mechanism. In the present study we measured the plasma concentration of acetylcholine and epinephrine that are parasympathetic and sympathetic neurotransmitter to discuss EA heart protective mechanism by evaluating autonomic nervous function.

Cellular metabolic disorders, systemic inflammatory damage caused by multiple organ dysfunction, and low blood vessel reactivity are important endogenous factors affecting shock treatment [13]. It is known that long-term high concentrations of catecholamine lead to lower sensitivity of adrenergic receptors [14]. At this point the high concentrations of catecholamine in human body fail to play the heart protection role by contracting blood vessels of skin, muscle, and abdominal organs but aggravate vascular low reactivity which further reduces the effective circulating blood volume. Without timely and effective treatment, patients will enter the refractory period, when intravascular coagulation and multiple organ failure occur which finally lead to death. In addition, the low reactivity of blood vessels has been proved to be key factor result in vasoactive drug failure. Therefore, improving autonomic function by suppressing overexcitation of sympathetic nerve is important to apply early prognosis and rescue on shock patients.

It has been proved that acupuncture affected a number of indicators related to autonomic nervous system, such as heart rate, pulse, heart rate variability (HRV), blood pressure, pupil size, skin conductance, and sympathetic and parasympathetic neurotransmitters. Current researchers hypothesize that acupuncture can balance parasympathetic and sympathetic nerve activities to play a therapeutic role by stimulating the peripheral nerve and body fluids. Our previous studies have shown that EA at ST36 could prevent severe scalds-induced gut ischemia, paralysis, and barrier dysfunction in rats with severe burn injuries. EA after atropine injection or cervical vagotomy failed to improve intestinal motility and mucosa blood flow suggesting that the mechanism of EA may be related to the activation of the cholinergic nerve pathway [15]. However, our clinical study showed that acupuncture improved both HRV and symptoms of anxiety patients. All of these results indicated that acupuncture is able to regulate autonomic function by suppressing overexcitation of sympathetic nerve and improving HRV, which is important to shock patients [16–18].

To sum up, when the patient has a hemorrhagic shock and the outside conditions do not allow infusion therapy, EA at ST36 could increase the parasympathetic excitability in the early stage of shock and restrain the excessive excitability of the sympathetic nerve in the shock. EA could prevent shock transfer to decompensation and irreversible stage, improving the protective function of the fluid resuscitation on heart in the early stage of shock.

## 2. Materials and Methods

### 2.1. Rat Model

60 Sprague Dawley male rats (7-8 weeks old, 230–250 g) were purchased from the Experimental Animal Center of Military Medical Sciences of the Chinese People's Liberation Army. The rats were housed in mesh cages in a room maintained at 25°C, illuminated with 12 : 12-hour light-dark cycles, and provided with standard rodent chow and water ad libitum. Rats were subjected to 45% blood volume loss. Surgeries were performed according to the method of Christian Shults [19]. After the experiments the animals were sacrificed. All animal experiments were approved by the Committee of Scientific Research of the First Hospital Affiliated to General Hospital of People's Liberation Army, Beijing, China (Institutional Animal Care and Use Committee (license number SCXK Beijing 2009/0007)), and were conducted in accordance with the National Institute of Health Guide for the Care and Use of Laboratory Animals.

### 2.2. Animal Grouping and Treatments

Experimental rats were randomly assigned to five groups (*n* = 12 each) after hemorrhagic shock.

#### 2.2.1. SHAM Group

Rats received sham hemorrhagic shock operation which only gives femoral arteriovenous catheter.

#### 2.2.2. EA Group

Animals underwent EA at ST36 points, which are located at the posterior and lateral side of the knee joint, 5 mm below capitulum fibulae [5], immediately after hemorrhagic shock. EA at ST36 was performed using an electroacupuncture apparatus (HANS, China, LH202H) as described before [5], with an intensity of 2 mA and a frequency of 2–100 Hz, for approximately 1.5 hours immediately after hemorrhagic shock. Rats were subjected to 45% blood volume loss.

#### 2.2.3. SEA Group

Rats received EA at nonchannel acupoint which is approximately 3 cm distal to the ST36 acupoint toward the tail and opposite to the knee joint. It is located over the semitendinosus muscle at 5 mm from the tail base. This nonacupoint is neither referred to in the acupoint map of rodents nor referred to close to any major nerve [19] immediately after injury. Other parameters were the same as those in the EA group.

#### 2.2.4. EA + DR Group

Delayed fluid resuscitation was performed after EA at ST36 points. EA parameters were the same as in the EA group.

#### 2.2.5. SEA + DR Group

Delayed fluid resuscitation was performed after SEA at ST36 points. SEA parameters were the same as those in the SEA group.

### 2.3. Caspase-3 Protein Activity Assay

100 *μ*l tissue lysis buffer per 3–10 mg tissue has been added. The pellet was homogenized on ice and then the mixture was transferred into a 1.5 ml EP tube followed by lyses on ice for another 5 min and centrifuged at 16,000–20,000*g* at 4°C for 15 min. The supernatant was transferred to a precool EP tube. According to the instruction of the caspase-3 activity test kit (Beyotime Biotech, Nantong, China), the mixture of sample and reagent was incubated at 37°C for 120 min. The absorptive value of caspase-3 protein was examined at A405nm. Caspase-3 activity was calculated by the standard curve and activity curve.

### 2.4. TUNEL Assay

The slide of heart tissue was dried for 5 min before being fixed with 4% paraformaldehyde for 20 min at room temperature and then the antigen was repaired. After that it was washed with phosphate buffered saline (PBST) for three times. The following steps were strictly guided by TUNEL analysis kit (Roche, Basel, Switzerland). As a result, the apoptotic nuclei were labeled with green fluorescence and observed under fluorescence microscope. Under the 400x microscope, 5 views were randomly picked at the infarct region and board region. The percentage of apoptosis cells as the index of apoptosis level of the heart tissue was calculated.

### 2.5. Neurotransmitter Analysis: Acetylcholine and Epinephrine

The blood sample was well homogenized after homogenizing centrifuge for 15 min at 5000 rpm and the supernatant was used for the assay immediately using the protocol mentioned in the BG (EPI-Epinephrine) ELISA kit (Blue Gene Biotech, China) and using ELISA micro plate, the yellow color developed was read immediately at 450 nm OD, after the stop solution was added.

### 2.6. Statistical Analysis

SPSS 13.0 statistical software was used, and all results were expressed as mean ± SD. One-way analysis of variance was used for comparison among all the groups, followed by the Student-Newman-Keuls test for comparison between two groups. Differences were considered to be statistically significant when *P* < 0.05.

## 3. Results

### 3.1. Effect of EA at ST36 on the Caspase-3 Activity of Cardiac Tissue

**Figure 1:**
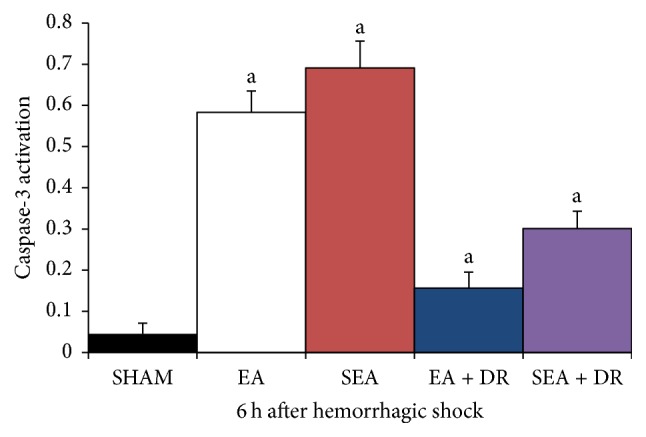
All groups indicated with “a” versus SHAM group, *P* < 0.05; EA group versus SEA group, *P* > 0.05. EA + DR group versus SEA + DR group within 6 h, *P* < 0.05.

Cardiac tissues was harvested to test caspase-3 activity after 45% blood loss for six hours. Hemorrhagic shock induced heart failure and increases the caspase-3 activity. Delayed fluid resuscitation was effective in decreasing the protein expression levels of caspase-3. The activity of caspase-3 in SEA group was significantly higher than other groups (*P* < 0.05). No significant differences were found between EA and SEA group (*P* > 0.05). The caspase-3 activity in EA + DR group was significantly lower than SEA + DR group (0.156 ± 0.039 versus 0.301 ± 0.042). The results are shown in Figure 1.

### 3.2. TUNEL Apoptotic Cells in Cardiac Tissues

**Figure 2:**
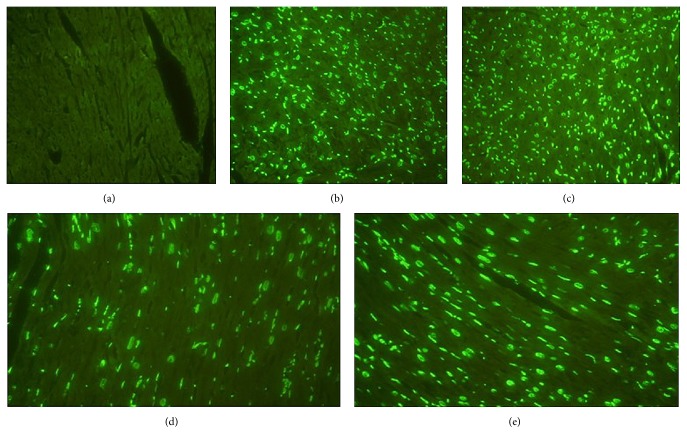
5 views (a–e) of the heart tissue under the 400x microscope which were randomly taken from different groups. The apoptotic nuclei were labeled with green fluorescence and observed under fluorescence microscope. (a–e) separately represent the groups of SHAM (a), EA (b), SEA (c), EA + DR (d), and SEA + DR (e).

**Figure 3:**
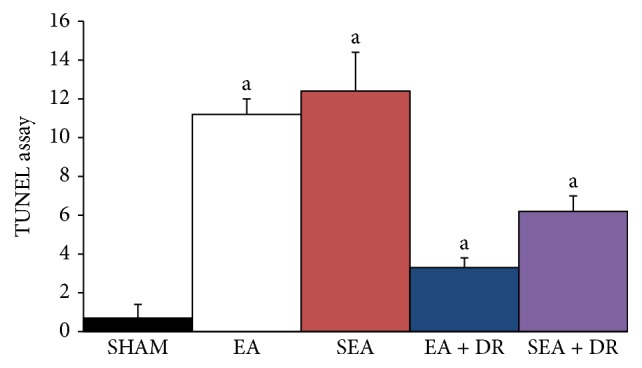
All groups indicated with “a” versus SHAM group, *P* < 0.05; EA group versus SEA group, *P* > 0.05. EA + DR group versus SEA + DR group within 6 h, *P* < 0.05.

DNA fragmentation in apoptosis is usually associated with structural changes in cellular morphology and can be examined using the TUNEL assay. The apoptotic cells and total cells were quantified using the TUNEL* assay* and DAPI staining, respectively, to view the apoptotic activity in cardiac tissues. Viewing images magnified ×400 (Figure 2), we observed that the left ventricles of the SEA group failed to be stained with the TUNEL assay. Groups of EA and SEA had a greater number of TUNEL-positive cardiac cells than those in the groups of SEA + DR and EA + DR. Moreover, the Image J software analyzed the number of apoptotic cells with one-way analysis of variance (ANOVA) statistical analysis, showing no significant differences between EA and SEA groups (*P* > 0.05). However, the left ventricles of the SEA + DR group had more TUNEL-positive cells than the EA + DR group (6.2 ± 0.8 versus 3.3 ± 0.5). The results are shown in Figure 3.

### 3.3. EA at ST36 Lowered the Plasma Level of Epinephrine

**Figure 4:**
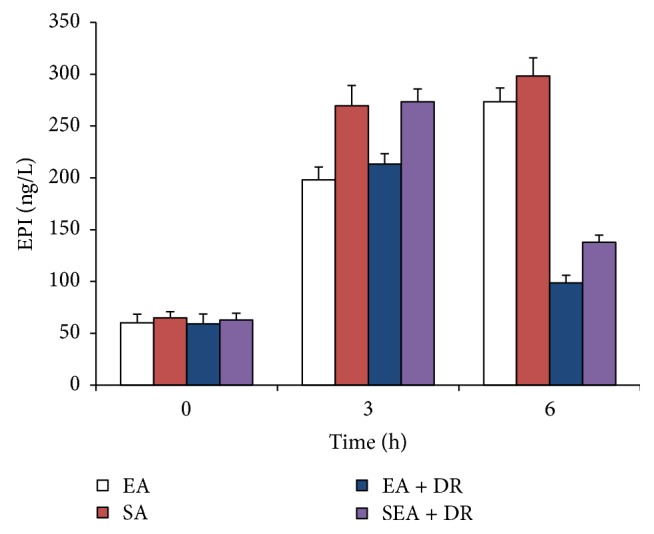
The level of plasma EPA was detected at 0, 3, and 6 h after shock. EA group versus SEA group within 3 h, *P* < 0.05. EA + DR group versus SEA + DR group within 6 h, *P* < 0.05.

The plasma level of epinephrine significantly increased after 45% blood loss for 3 h and 6 h (Figure 4). Delayed fluid resuscitation attenuates the increase of epinephrine. However, EA at ST36 played the same role as delayed fluid resuscitation. The plasma level of epinephrine in EA group was significantly lower than that in the SEA group after 45% blood loss for 3 hours; after hemorrhagic shock for 6 h, epinephrine level of EA + DR group was significantly lower than in the SEA + DR group (98.6 ± 7.4 ng/L versus 137.8 ± 6.9 ng/L; *P* < 0.05).

### 3.4. EA at ST36 Increased the Plasma Level of Acetylcholine

**Figure 5:**
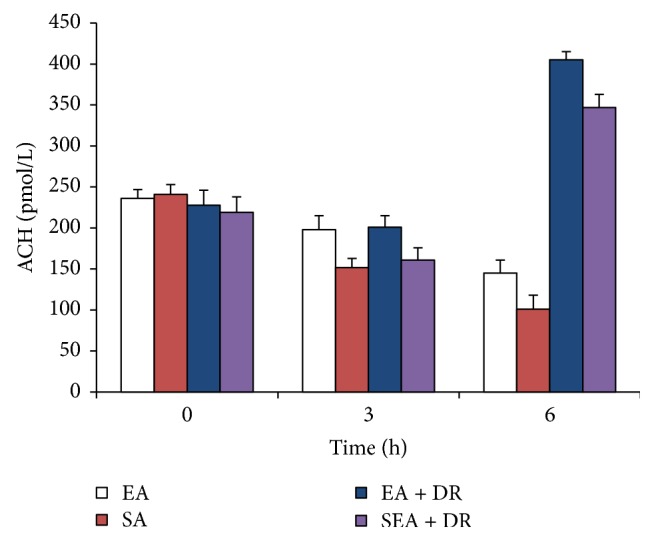
The level of plasma ACH was detected at 0, 3, and 6 h after shock. EA group versus SEA group within 3 h, *P* < 0.05. EA + DR group versus SEA + DR group within 6 h, *P* < 0.05.

The plasma level of acetylcholine in EA group was significantly higher than in the SEA group after shock for 3 hours (*P* < 0.05): after hemorrhagic shock for 6 h, acetylcholine level of EA + DR group was significantly higher than SEA + DR group (405 ± 10 pmol/L versus 347 ± 16 pmol/L; *P* < 0.05). The results are shown in Figure 5.

## 4. Discussion

Both plasma epinephrine and acetylcholine are neurotransmitters secreted by nerve terminals. The plasma concentration of epinephrine mainly reflects the excitability of the sympathetic nervous system. The concentration of acetylcholine in plasma may reflect the activity of vagus nerve. In this study, the concentration of epinephrine and acetylcholine in the plasma of rats at different time points was quantitatively measured with the excitability of sympathetic and parasympathetic nerves. The results showed that the plasma epinephrine levels of rats in each group went up significantly compared with those in 3 h and 6 h after blood loss (*P* < 0.05). 3 h and 6 h after blood loss, the level of plasma adrenaline in the EA group was significantly lower than that in sham acupuncture group (*P* < 0.05). 6 h after shock, the plasma adrenal concentration in the EA + DR group was significantly lower than that in the SEA + DR group. These indicate that EA at ST36 immediately after shock can effectively inhibit the increase of plasma adrenal gland concentration in rats. It is indicated that EA at ST36 can effectively enhance the therapeutic effect of delayed resuscitation and inhibit the excessive adrenaline level in rats. The plasma level of acetylcholine in EA group was significantly higher than SEA group after shock for 3 hours (*P* < 0.05); after hemorrhagic shock for 6 h, acetylcholine level of EA + DR group was significantly higher than SEA + DR group (*P* < 0.05). The current study for early treatment of hemorrhagic shock focuses on adequate fluid resuscitation which is very important for vital organ function [20]. This study indicated that EA at ST36 played the same protective role as fluid resuscitation. The above data demonstrated that EA could effectively activate the parasympathetic nerve by promoting acetylcholine release and suppressed sympathetic overexcitement. As a result immediately EA at ST36 after the blood loss enhanced the protective effect of delayed resuscitation on the cardiac tissue of hemorrhagic shock rats.

The function and structure of cardiac tissue are one of the key factors affecting prognosis of hemorrhagic shock [21]. The results of the present study indicated that hemorrhagic shock contributed organ cell apoptosis, especially myocardial cell apoptosis. Caspase-3 is the major regulator in the downstream apoptosis signaling pathway. Either extracellular receptor mediated apoptosis or intracellular mitochondrial mediated apoptosis ultimately induced the cell death through activating substrate degradation by caspase-3 [18]. DNA fragmentation in apoptosis is usually associated with structural changes in cellular morphology and can be examined using the TUNEL assay. Therefore, this study tested both caspase-3 activation and positive rate of TUNEL to detect cardiac protecting effects of EA on hemorrhagic shock rats. We observed green fluorescence labeled apoptotic nuclei under the 400x microscope. The results showed that there were no apoptotic cells found in the SHAM group, but a great number of the apoptotic cells were observed after 6 hours of hemorrhagic shock in other groups. However, the SEA + DR and EA + DR group had fewer apoptotic cells than EA and SEA group. The caspase-3 activity and positive rate of TUNEL in EA + DR group were significantly lower than other hemorrhagic shock groups. All of the above results showed that delayed fluid resuscitation significantly inhibited myocardial apoptosis after hemorrhagic shock. Immediately EA treatment after the blood loss enhanced the protective effect of delayed resuscitation on the cardiac tissue of hemorrhagic shock rats.

After blood loss for 6 hours, the caspase-3 activity of EA + DR group was significantly lower than SEA + DR group and the positive rate of TUNEL in EA + DR group was also significantly lower than SEA + DR. As we mentioned before, both caspase-3 activity and TUNEL-positive rate could be used to detect the apoptotic myocardial cell. All data suggested that EA treatment inhabited myocardial cell apoptosis of hemorrhagic shock rats after applying delayed fluid resuscitation. At the same time, we also found that acetylcholine level of EA + DR group was significantly higher than SEA + DR group. However, epinephrine level of EA + DR group was significantly lower than SEA + DR group. As a result, the plasma concentration of epinephrine mainly reflects the excitability of the sympathetic nervous system and the concentration of acetylcholine in plasma reflects the activity of vagus nerve. We suggested that EA treatment before the delayed fluid resuscitation could protect the heart of the rats with hemorrhagic shock by regulating the autonomic nervous system.

As mentioned before, there are some other animal experimental studies using EA and moxibustion in mice and rats to investigate the protective role on heart. Shen et al. [9] investigated ten inbred mice. Neiguan (PC 6) and Fenglong (ST 40) were punctured in the acupoint group; intragastric administration of simvastatin was applied in the medication group. After 8 weeks of treatment, the changes of different parameters were quantified. The rising range of blood lipid was obviously decreased in the acupoint group and medication group, and thickness of ventricular wall was reduced [9].

In another study [10] preventative moxibustion was applied to Zusanli (ST 36) and Guanyuan (CV 4) for 5 min, once daily for 3 weeks. The apoptotic cardiomyocytes were detected by light microscope after terminal deoxynucleotidyl transferase mediated dUTP-biotin nick end labeling (TUNEL staining), and the ultrastructure of cardiomyocytes was observed by using transmission electron microscope. Compared with the blank control group, the rates of the apoptotic cardiomyocytes were considerably increased. In mice experiencing preventative moxibustion, the injured state of the cardiomyocytes included dissolved myofilaments of myocardial fibers, disorder of arrangement and increased interspace of myofilaments, and decreased number of partial myocardial bundles, and the increase of matrix electron density was relatively milder in comparison with their individual exercise model groups. The authors stated that preventative moxibustion may reduce moderate-intensity exercise and exhausted exercise induced apoptosis of cardiomyocytes in mice and lessen myocardial injury [10].

Xie et al. [11] reported in 2017 the different effects of EA of Neiguan (PC 6) grouping with other acupoints on myocardial ischemia injury, so as to provide evidence for clinical acupoint formulas. Forty Wistar rats were investigated using different kind of EA. The researchers from China concluded from their animal experimental study that EA can prevent myocardial ischemic injury [11].

In our study it is interesting that the caspase-3 activity and positive rate of TUNEL were insignificant between EA and SEA groups. But we detected significant differences of plasma epinephrine and acetylcholine between EA and SEA groups within 3 h. So it would take some time for EA to perform heart protecting effects. As a result, it is necessary to do EA treatment immediately after the shock. As we all know, intravenous fluid resuscitation is usually difficult to administer in some environments. In recent years, we have been working on alternative methods to solve the complications caused by delayed fluid resuscitation and we have proved that EA at ST36 can significantly protect the intestinal barrier integrity and improve organ function and survival rate after fatal hemorrhagic shock in rats [7, 8]. Recent studies showed that electrical stimulation of the vagus nerve could protect alleviation of inflammatory injuries and remote organs of animals following burn injuries by activation of the cholinergic anti-inflammatory pathway [22]. But electrical stimulation of the vagus nerve is not easy and safe to apply in patients. In this study we apply immediately EA as an alternative treatment to regulate autonomic nerve which turned out to be safe and effective.

Autonomic dysfunction can induce significant and heterogeneous changes of atrial electrophysiology and induce atrial tachyarrhythmia [23]. It is well known that autonomic nervous system consists of the sympathetic nervous system and the parasympathetic nervous system. Both of these systems can stimulate and inhibit effectors. However, the two systems work in opposition where one system stimulates an organ and the other inhibits the organ. Working in this fashion, each system prepares the body for a different kind of situation.

When body suffers from hemorrhagic shock, the sympathetic part becomes overactive and the parasympathetic part is suppressed, contributing to tachyarrhythmia. However, 45% blood loss and tachyarrhythmia lead to heart failure. If acupuncture could regulate autonomic nervous system by suppressing the sympathetic overexcited part and activate the parasympathetic part, it could protect hemorrhagic shock rats from heart failure.

There are several limitations in this study. All of our measures were evaluated with the rats under anesthesia, which might be different between human and animals. Meanwhile, more* in vitro *researches are needed to clarify the mechanism of heart protecting effect of EA at ST36. Besides, the model we used in this study is subjected to withdrawing about 45% of the calculated TBV and such a large volume may be lethal and cause severe damage to the animals.

## 5. Conclusion

Immediately EA at Zusanli after blood loss apparently enhanced the protective effect of delayed resuscitation on the cardiac tissue of hemorrhagic shock rats. Its mechanism is possibly related to the improvement of the autonomic nervous function.
